# Working Alliance Inventory for Online Interventions-Short Form (WAI-TECH-SF): The Role of the Therapeutic Alliance between Patient and Online Program in Therapeutic Outcomes

**DOI:** 10.3390/ijerph17176169

**Published:** 2020-08-25

**Authors:** Rocío Herrero, Mª Dolores Vara, Marta Miragall, Cristina Botella, Azucena García-Palacios, Heleen Riper, Annet Kleiboer, Rosa Mª Baños

**Affiliations:** 1 Polibienestar Research Institute, University of Valencia, 46022 Valencia, Spain; ro.herrero.09@gmail.com (R.H.); m.dolores.vara@uv.es (M.D.V.); banos@uv.es (R.M.B.); 2 CIBERObn Physiopathology of Obesity and Nutrition, Instituto de Salud Carlos III, 28029 Madrid, Spain; botella@uji.es (C.B.); azucena@uji.es (A.G.-P.); 3 Department of Basic and Clinical Psychology and Psychobiology, Faculty of Health Sciences, Jaume I University, 12071 Castellon de la Plana, Spain; 4 Department of Clinical, Neuro and Developmental Psychology, Vrije Universiteit, 1081-BT Amsterdam, The Netherlands; h.riper@vu.nl (H.R.); a.m.kleiboer@vu.nl (A.K.); 5 Department of Research and Innovation GGZ InGeest, Amsterdam University Medical Center, 1081-HJ Amsterdam, The Netherlands; 6 Department of Personality, Evaluation and Psychological Treatment, Faculty of Psychology, University of Valencia, 46010 Valencia, Spain

**Keywords:** therapeutic alliance, online interventions, therapeutic outcomes, satisfaction with the treatment

## Abstract

Background: Therapeutic alliance (TA) between the patient and therapist has been related to positive therapeutic outcomes. Because Internet-based interventions are increasingly being implemented, a tool is needed to measure the TA with Internet-based self-guided programs. The Working Alliance Inventory for online interventions (WAI-TECH-SF) was adapted based on the WAI Short Form (Hatcher & Gillaspy, 2006). The objectives of this study were: (1) to analyse the psychometric properties of the WAI-TECH-SF; (2) to explore the differences in the WAI-TECH-SF scores according to different categories of the sample; and (3) to analyse whether the WAI-TECH-SF can predict therapeutic outcomes and satisfaction with the treatment. Methods: 193 patients diagnosed with depression were included and received blended Cognitive-Behavioural Therapy. Measures of preferences, satisfaction, and credibility about the treatment, TA with the online program, depressive symptoms, and satisfaction with the treatment were administered. Results: An exploratory factor analysis revealed a one-dimensional structure with adequate internal consistency. Linear regression analyses showed that the WAI-TECH-SF predicted changes in depressive symptoms and satisfaction with the treatment. Conclusions: WAI-TECH-SF is a reliable questionnaire to assess the TA between the patient and the online program, which is associated with positive therapeutic outcomes and satisfaction with the treatment.

## 1. Introduction

Evidence shows that the therapeutic alliance (TA) (also called the working alliance) has a relevant influence on therapeutic outcomes [1]. Several meta-analyses have found that TA is moderately associated with better treatment outcomes in face-to-face therapy [1,2], regardless of the therapeutic framework or patient characteristics, among other aspects [3]. One of the most widely used definitions of TA was proposed by Bordin [4], who considered the alliance to be a general factor with three interrelated components: (a) the degree of mutual trust, collaboration, and acceptance between the therapist and the patient (i.e., the bonds); (b) the agreement between patient and therapist about specific tasks or activities (i.e., the tasks); and (c) the agreement about the therapeutic objectives (i.e., the goals).

Currently, the ways of delivering therapeutic interventions are changing. The need for psychological support is growing, and the dominant model of psychotherapy from past centuries—individual, face-to-face, and long-lasting—is not likely to fulfil this need [5]. New forms of treatment delivery are emerging to face the challenge of providing well-established interventions that can reach a wider population. This situation has promoted the development of interventions that require less therapist involvement, such as Internet-based Interventions (IBIs) [6], either self-guided or hybrid therapeutic approaches (blended treatments) where face-to-face sessions are combined with online therapy [7]. Well-established evidence shows that interventions fully or partially delivered through the Internet (especially those based on Cognitive Behavioural Therapy (CBT)) are effective in treating different psychological disorders [8,9,10] and can be as effective as face-to-face therapy [11,12]. Therefore, IBIs are attractive and useful strategies to be applied by healthcare professionals in clinical settings [13]. Nevertheless, different questions have arisen about the therapeutic process, such as what happens to the TA when there is no direct contact with a therapist or when this contact is scarce [14].

The research triggered by this question has focused on the development and validation of measures that make it possible to assess the TA in IBIs and study of the role of the TA in predicting the therapeutic outcomes. To measure the TA in therapies supported by technological tools, most studies have used an adapted version of the Working Alliance Inventory (WAI) [15], or its short form (WAI-SF) [16,17]. In general, evidence supports the relevance of the TA in IBIs, because similar TA scores have been found for face-to-face therapy and IBIs [3,14,18,19,20], as well as similar moderate effect sizes for the relationship between TA and therapeutic outcomes [3,21]. In this regard, Clarke et al. [22] conducted a qualitative and quantitative study to examine the TA in the context of a self-guided intervention, and they found that TA is high even when the intervention does not involve human support. In addition, the study highlighted that a positive TA, in terms of feeling a meaningful connection and working collaboratively, is relevant for engaging with the intervention. In this study, they did not find a relationship between TA and therapeutic outcomes. Nonetheless, a systematic review showed an effect of TA on anxiety and depressive outcomes, pointing out that higher levels of TA were related to better clinical outcomes [23]. Recently, Gómez-Penedo et al. [24] explored the reliability and validity of the WAI-SF in guided IBIs, taking into account the relational aspects involved in this type of intervention (e.g., therapist support, online program). In this validation, the bond subscale was adapted to refer to the acceptance and trust between the patient and the therapist who supported him/her in the online program. Results of the WAI for guided Internet interventions (i.e., WAI-I) showed a two-factor solution (“tasks-goals” and “bonds”) with adequate internal consistency and external validity in a sample of patients with mild to moderate depression. Moreover, patients with higher scores on TA were more satisfied with the intervention after the treatment.

The approach used to carry out the adaptation of the WAI questionnaire in previous studies consisted of following Bordin’s classic conceptualization [4], considering the three dimensions of TA (bond, tasks, and goals) and making slight modifications in the statements [25,26]. This approach has been questioned by some authors, who suggest that the definition of TA is grounded in the specific characteristics of face-to-face therapy and, therefore, is not necessarily the same in other formats [27,28]. In fact, in the WAI adapted to measure the TA between the patients and the virtual environment, where the word “therapist” was replaced by “virtual environment”, the three dimensions proposed by Bordin [4] did not arise in the exploratory factor analysis, and only one general dimension was found [25]. By contrast, Kiluk et al. [29] conducted an adaptation of the 36-item original version of the WAI called WAI-Tech, which was designed to measure the TA between cocaine-dependent patients and the online program, and it showed similar psychometric properties to the original scale (i.e., the WAI). In this adaptation, items were slightly adapted by replacing “therapist” with “online program”, and items corresponding to the bond subscale were reworded to preserve comprehension. Results showed lower scores on the bond subscale, and the total scores on WAI-Tech were not associated with the change in therapeutic outcomes. However, findings are limited due to the small sample size and the absence of factor analysis to analyse the psychometric properties in this study.

Thus, the TA developed by the patients in IBIs and, specifically, in blended Cognitive-Behavioural Therapy (CBT) (i.e., combining individual face-to-face sessions with online intervention modules) has not been completely understood. The present study was conducted in the context of a European project called “e-Compared”, in which previous findings found that only therapist-rated TA (but not patient-rated TA) was predictive of changes in depression scores during a blended treatment in a sample of 73 patients [30]. Thus, it seems that technology is a third factor in the relationship between patient and therapist, which adds more complexity to this relationship. Hence, the need to measure not only the relationship between the patient and the therapist, but also between the patient and the technology, is undeniably relevant due to the growing emergence of IBIs.

Previous studies have found that individuals have the ability to form a bond and be open with an online application [28]. However, it is still important to develop a reliable questionnaire and explore whether the three-dimensional structure proposed originally for face-to-face therapy is also maintained in the TA with the online program in self-guided IBIs where there is hardly any interaction with a therapist or person supporting the intervention. To do so, an adaptation of the WAI-SF to measure the TA with the online program was carried out. An exploratory factor analysis was conducted in order to avoid determining the psychometric structure a priori, given the controversial structure of TA when technologies are involved (e.g., the structure was uni-dimensional in Miragall et al. [25]; or bi-dimensional in Gómez-Penedo et al. [24]). In addition, other potential variables influencing the TA with the online program were explored, as well as the capacity of the TA with the online program to predict therapeutic outcomes and satisfaction with the treatment. The study was conducted in a sample of depressive patients who were receiving a self-guided IBI in the context of the National Health Systems of different European countries.

Hence, the aims of this study were: (1) to analyse the psychometric structure of the WAI-TECH-SF, a questionnaire designed to assess the TA between the patient and the online program in a self-guided IBI; (2) to explore whether there are differences in WAI-TECH-SF scores based on sex, age-range, level of education, initial severity of depression, preference for any of the treatments offered, and expectations about and credibility of the treatment; and (3) to explore whether higher WAI-TECH-SF scores predict the therapeutic outcomes (i.e., change in depressive symptom scores) and satisfaction with the treatment.

## 2. Materials and Methods

### 2.1. Participants

One-hundred and ninety-three patients took part in this study (ages ranging from 19 to 69 years old: *M* = 40.44 years old; *SD* = 12.79; 64.2% women). Patients were recruited as part of the clinical trial conducted in the e-Compared Project (EU-HEALTH.2013 N.603098). The sample was composed of European citizens diagnosed with depression in either primary or specialized care. Regarding their nationalities, 38.2% of the sample were from Germany, 16.2% from Sweden, 12.0% from Spain, 9.8% from France, 8.4% from the Netherlands, 6.3% from the UK, and 2.1% from Switzerland. Patients were excluded if they were under 18 years old, had serious psychiatric comorbidity, or did not have access to a computer or the Internet. All the patients were diagnosed with depression using the MINI. In terms of their symptoms, 10.9% of the sample showed mild symptoms of depression, 33.2% showed moderate symptoms, 36.8% showed moderate–severe symptoms, and 19.2% showed severe depressive symptomatology. In addition, 50.8% of the sample had some suicidal risk, and 61.1% of the sample had a comorbid diagnosis, such as panic disorder, agoraphobia, or social phobia. Regarding their educational level, 56.0% of the sample had a high educational level, 31.6% had a medium educational level, and 12.4% had a low educational level. All participants were informed about the study and gave their informed consent before the beginning of the trial, in accordance with the Declaration of Helsinki. The study was approved by the corresponding ethical committee in each country: (a) France: Comité de protection des personnes, Ile de France V (15033-n° 2015-A00565-44); (b) Germany: Ethik Kommison DGPsychologie, Universitat Trier (MB 102014); (c) The Netherlands: METC VUMC (2015.078); (d) Poland: Komisja ds. Etyki Badan Naukowych (10/2014); (e) Spain: Comision Deontologica/Comite Ético de Investigacion en Humanos de la Universidad de Valencia (H1414775276823); (f) Sweden: Regionala etikprovningsnamnden (2014/428-31); (g) Switzerland: Kantonale Ethikkomission Bern (001/2015); (h) UK: NRES Committee London-Camden and King’s Cross (15/LO/0511). All participants received a blended CBT (bCBT) for depression.

### 2.2. Intervention

All the e-Compared project interventions combined individual CBT delivered through face-to-face sessions and online sessions [31]. The interventions received by patients had some variations across the countries, but followed common guidelines [31]. In this regard, the ratio between the number of face-to-face sessions and the number of online modules varied across countries, but at least 1/3 of the sessions were face-to-face (i.e., between 3 and 10 sessions), and at least 1/3 were online (i.e., between 6 and 10 sessions). As a minimum, the bCBT included modules of psychoeducation, cognitive restructuring, behavioural activation, and relapse prevention. In addition, each country site was able to include additional components, such as mindfulness, coping skills training, or problem solving, but these additional components could not make up more than a quarter of the total intervention. Face-to-face sessions were provided by: (1) licensed CBT therapists in mental health care; (2) CBT therapists in training under the supervision of an experienced licensed CBT therapist in mental health care; (3) a licensed psychologist with a CBT orientation in primary care; or (4) psychologists in training under the supervision of a licensed psychologist with a CBT orientation in primary care. All of them were trained in how to deliver the blended treatment. Each face-to-face session lasted around 20–60 min (i.e., 45–60 min in specialized care and 20–45 min in primary care), while the online session lasted for as long as the patients took to read each session. A summary of the intervention components and the online vs. face-to face ratio are shown in Table 1.

### 2.3. Measures

Working Alliance Inventory applied to Internet (WAI-TECH-SF) is an adaptation of the WAI-SF [16] elaborated by the authors. It is a 12-item self-report questionnaire designed to assess the TA with the online program in a self-guided IBI, with responses rated on a seven-point Likert scale, ranging from 1 (never) to 7 (always). The questionnaire was designed to cover the same structure as the original scale, with three dimensions: (1) therapeutic goals (items 1, 2, 8, 10), (2) tasks (items 4, 6, 10, 11), and (3) bonds (items 3, 5, 7, 9). The total score ranges from 12 to 84. The mean and standard deviation for this sample were *M* = 57.84 and *SD* = 16.39. Details about its adaptation appear in the “Procedure” section. The questionnaire was administered at post-assessment.

Patient Health Questionnaire-9 (PHQ-9; [32]) is a nine-item mood module that can be used to screen and diagnose patients with depressive disorders. It is based directly on the criteria for major depressive disorder in the Diagnostic and Statistical Manual of Mental Disorders (4th ed.) [33] and its accuracy for screening to detect major depression has been demonstrated [34]. The nine items are each scored on a 0–3 scale, with the total score ranging from 0–27 and higher scores indicating more severe depression. The means and standard deviations for this sample were M = 15.50 and SD = 4.63 (pre-assessment), and M = 9.05 and SD = 5.35 (post-assessment). The PHQ-9 has been shown to have good psychometric properties [35]. The questionnaire was administered at pre- and post-assessment. In this study, Cronbach’s alphas ranged from 0.73 to 0.87.

International Neuropsychiatric Interview (MINI 5.0; [36]) is a structured diagnostic interview based on the Diagnostic and Statistical Manual of Mental Disorders (DSM-IV) and on International Classification of Diseases (ICD-10) criteria. The MINI has been translated into 65 languages and is used for both clinical and research practices. The full MINI. 5.0, with the exception of Anorexia Nervosa, Bulimia Nervosa, and Antisocial Personality Disorder, was used to provide a diagnosis at pre-assessment.

Preference for Treatment Questionnaire (ad-hoc instrument) was used to assess participants’ treatment preference from the options of bCBT, TAU, or no preference. Specifically, the following question was asked: “If you had the chance to choose your depression treatment, which one would you prefer to receive?”

Credibility and Expectancy Questionnaire (CEQ; [37]) was used to assess the prior predisposition of patients to the proposed intervention. The scale consists of six items divided into two factors: expectancy (with three questions rated on a 10-point scale, ranging from 1 to 9) and credibility (with one question rated on a 10-point scale and two questions rated on a 1–100% scale). The means and standard deviations for this sample were *M* = 17.59 and *SD* = 4.92 (in a scale ranging from 3 to 27) and *M* = 19.32 and *SD* = 5.15 (on a scale ranging from 3 to 27) for expectancy and credibility, respectively. In this study, Cronbach’s alphas were 0.86 for expectancy and 0.72 for credibility.

Client Satisfaction Questionnaire (CSQ-8; [38]) was used to assess patients’ satisfaction with the treatment. This questionnaire has been translated into multiple languages, and it is used to measure global patient satisfaction. The questionnaire consists of eight items rated on a four-point scale, with total scores ranging from 8 to 32. The mean and standard deviation for this sample were *M* = 25.39 and *SD* = 5.00. The questionnaire was administered at post-assessment. In this study, Cronbach’s alpha was 0.92.

### 2.4. Procedure

An adaptation of the patient version of the WAI-SF [16] was carried out following the recommendations of Hambleton and Patsula [39]. Thus, the purpose of the WAI-TECH-SF is to measure agreement about goals, tasks, sense of trust, comfort, and bonding between the patient and the “online program”. To this end, the sentences on the current scale were kept as similar as possible to the originals, but “my therapist” or therapy was replaced with “online program”. Items are displayed in Table 2.

Once the WAI-TECH-SF had been adapted, it was applied in the context of the e-Compared European project to the participants receiving the bCBT. All the patients in the project were recruited in the National Health Systems of the countries involved, in either primary or specialised care. Their status was assessed with the MINI interview, performed by a clinical psychologist. If patients met the inclusion criteria, they were allocated to one of two conditions: bCBT or Treatment as Usual (TAU) (for more details about the trial, see Kleiboer et al. [31]). All participants filled out the PHQ-9 questionnaire to assess the severity of their depressive symptoms and their preference for the intervention (“blended”, “TAU”, or “no preference”), and the CEQ scale was used to assess the patients’ expectations and credibility with regard to the intervention offered. For the purposes of the current study, only participants allocated to the bCBT condition were taken into account, given that those in the TAU condition did not receive any therapeutic support online. Once patients had finished the intervention, they were assessed again on their depressive symptoms, their satisfaction with the treatment through the CSQ scale, and their TA with the self-guided IBIs using the WAI-TECH-SF.

### 2.5. Data Analyses

All statistical analyses were performed using the SPSS v.26 (IBM Corp, Armonk, NY, USA). The percentage of missing values in the WAI-TECH-SF, PHQ-9, and CEQ scores ranged from 0% to 1.6%. After testing that the values were missing at random using Little’s MCAR test (*p* > 0.05), they were imputed using the Expectation–Maximization Algorithm method [40]. Then, several analyses were carried out. First, to analyse the psychometric properties of the WAI-TECH-SF, skewness and kurtosis were analysed to check the normality of the data [41]. Kaiser-Meyer-Olkin (KMO), and Barlett’s Test of Sphericity was used to ensure the suitability of the data for performing an Exploratory Factor Analysis (EFA). Parallel Analysis [42] was applied using a macro for SPSS [43] to determine the number of factors retained in the EFA. Then, to explore the factor structure of the WAI-TECH-SF, an EFA was conducted using a Maximum Likelihood estimation extraction method because the data were normally distributed [41]. Internal consistency of the total score was assessed using Cronbach’s alpha coefficient [44].

Second, preliminary analyses were conducted to ensure that relevant assumptions of t-tests, ANOVAs, and simple/multiple regression (i.e., normality, linearity, homoscedasticity, and absence of multicollinearity) were met. Third, independent-samples t-tests and one-way ANOVAs were performed to find out whether there were significant differences in the WAI-TECH-SF scores based on sex, age range (18–34 vs. 35–49 vs. > 50), level of education (low vs. medium vs. high), initial severity on PHQ scores (mild vs. moderate vs. moderate–severe vs. severe), preference for any of the treatments offered (no preference vs. blended vs. TAU), and expectations and credibility towards the treatment. Expectations and credibility scores were categorized as low (Mean–1 Standard deviation), medium (Mean), and high (Mean + 1 Standard deviation). *T*-values are reported as absolute values.

Fourth, two simple linear regression analyses (using the enter method) were carried out to study whether the WAI-TECH-SF scores predicted the changes in PHQ scores and satisfaction with the treatment. PHQ scores were calculated using the differences between post- and pre-assessment scores (Post–Pre). Thus, positive values indicated an increase in depression symptoms, whereas negative values indicated a decrease in depression symptoms.

Finally, a power analysis was conducted to determine whether the present study was adequately powered with our sample size (*N* = 193) (the sample size of this study was initially calculated for testing the hypothesis that bCBT was not inferior to the TAU condition on the primary clinical outcome (i.e., symptoms of depression at 3 months after baseline) (see Kleiboer et al. [31]), but not for the secondary outcomes and analyses). Using G*power v. 3.1.9.743 (Heinrich-Heine-Universität, Düsseldorf, Germany), we calculated power for: (1) an omnibus *F*-test “Fixed effects, one-way”; (2) a *t*-test “Differences between two independent means”; and (3) an omnibus *F*-test “Lineal multiple regression: fixed model, R^2^ deviation from zero”. An effect size of f = 0.20 or f^2^ = 0.12 was used because there is still limited data in this field and *d* = 0.40 is a standard in Psychology, according to Brysbaert [45]. Results indicated that the current study had 69.38% and 62.68% power for one-way ANOVAs with three and four groups, respectively, 75.49% for the *t*-test, and 99.77% for the regression analyses with one predictor to detect a medium effect size at *p* < 0.05.

## 3. Results

### 3.1. Psychometric Properties of WAI-TECH-SF

A random percentage of missing values was found, with Little’s MCAR test, χ^2^(33) = 14.27, *p* = 0.998, ranging from 0 to 1.6% per item. Consequently, items’ missing values were imputed using the Expectation–Maximization Algorithm method [38]. The sample’s normality was assumed because skewness values were <|2|, and kurtosis values were <|7| [46,47] (see Table 2). The KMO value was (0.96), and the Barlett’s Test of Sphericity value, χ^2^(66) = 2587.26, *p* < 0.001, showed that it was appropriate to perform a factor analysis. Regarding the number of factors to extract, Parallel Analysis [40] showed that one factor had to be retained because only one factor had an eigenvalue (raw data eigenvalue = 9.08) greater than the eigenvalue at the 95th percentile for randomly generated data (95th percentile eigenvalue = 1.53) [48]. Factorial rotation with one dimension was performed using the Maximum Likelihood extraction method, which showed that one dimension explained 73.49% of the total variance. The factorial solution showed that all the items had minimum factor loadings and communalities above ≥0.30 (see Table 2). Cronbach’s alpha coefficient for the WAI-TECH-SF was high for the overall scale (α = 0.97). We analysed the item–total correlation, and the exclusion of any item increased the alpha value for the overall scale.

### 3.2. Differences in WAI-TECH-SF Scores According to Socio-Demographic Variables, Initial Severity on PHQ Scores, Preference for the Treatment Offered, and Expectations and Credibility towards the Treatment

Table 3 shows the means and standard deviations of the WAI-TECH-SF scores according to sex, age-range, level of education, initial severity on PHQ scores, preference for any of the treatments offered, and expectations and credibility towards the treatment.

Independent-sample *t*-tests and one-way ANOVAs showed that there were no significant differences in the WAI-TECH-SF scores based on sex, age-range, initial severity of depression, preferences for any of the treatments offered, expectations about the treatment, and credibility of the treatment. However, there were significant differences in the WAI-TECH-SF scores based on the level of education. Patients with high (vs. low) education levels achieved higher scores on the WAI-TECH-SF, *p* = 0.042.

#### An Exploratory Multiple Regression Analysis: Socio-Demographic Variables, Initial Severity on PHQ Scores, Preference for the Treatment Offered, and Expectations and Credibility towards the Treatment as Predictors of WAI-TECH-SF Scores

Given the number of potential predictor variables of the WAI-TECH-SF, we also carried out a stepwise linear regression in order to analyse the explained variance by each variable. To do so, age, expectations, and credibility towards the treatment were maintained as continuous variables. Categorical predictor variables (i.e., level of education, initial severity of depression, and preference for any of the treatments offered) were transformed into dummy-coded variables. The reference category was “low” (vs. “medium and high”) for level of education, “mild” (vs. moderate, moderate-severe and severe) for initial severity of depression, and “no preference” (vs. blended and treatment as usual) for preference for any of the treatments offered.

Results of this regression analysis showed that two models were significant. The first model included level of education (*β* = 0.180, *t* = 2.462, *p* = 0.015) as a positive significant predictor of the WAI-TECH-SF scores. This model was significant, *F*(1,181) = 6.059, *p* = 0.015, explaining 2.7% of the variance.

The second model included level of education (*β* = 0.205, *t* = 2.798, *p* = 0.006) and age (*β* = 0.167, *t* = 2.279, *p* = 0.024) as positive significant predictors of the WAI-TECH-SF scores. This model was significant, *F*(2,181) = 5.696, *p* = 0.004, explaining 4.9% of the variance.

### 3.3. Predictive Models: Are Changes in PHQ Scores and Satisfaction with the Treatment Predicted by WAI-TECH-SF Scores?

The model where changes in PHQ pre-post intervention scores were predicted by WAI-TECH-SF scores was statistically significant, *F*(1,188) = 14.42, *p* < 0.001, explaining 6.7% of the variance. Higher scores on the WAI-TECH-SF predicted a greater decrease in depression symptoms.

Similarly, the model in which satisfaction with the treatment was predicted by the WAI-TECH-SF scores was statistically significant, *F*(1,187) = 185.53, *p* < 0.001, explaining 49.7% of the variance. Higher scores on the WAI-TECH-SF predicted higher scores on satisfaction with the treatment (see Table 4).

## 4. Discussion

The objectives of this study were: (1) to explore the psychometric structure of a questionnaire (i.e., the WAI-TECH-SF) designed to assess the TA with an online program in a self-guided IBI and CBT program in a sample of depressive patients in the context of the National Health Systems of different European countries; (2) to analyse whether there were differences in the WAI-TECH-SF scores based on several socio-demographic variables, initial symptoms of depression, preference for any of the treatments offered, and expectations and credibility towards the treatment; and (3) to study the capacity of the WAI-TECH-SF scores to predict the therapeutic outcomes (i.e., changes in depressive symptoms) and satisfaction with the treatment.

With regard to the psychometric properties of the WAI-TECH-SF, a unidimensional structure emerged in the EFA that accounted for 73.49% of the explained variance. All the factors had high factor loadings, and the overall scale had excellent internal consistency. This unidimensionality is in line with the structure found in the validation of the WAI applied to virtual and augmented reality (WAI-VAR, [25]). However, this structure is inconsistent with the three-dimensional structure of Bordin’s [4] theory and the original validation of the WAI-SF carried out by Hatcher and Gallispy [16] to measure TA in the face-to-face context, distinguishing three separate factors: tasks, goals, and bonds. Nevertheless, the structure of this questionnaire is controversial because a bi-factorial structure has also been found in other validations of the WAI, such as in Gómez-Penedo et al. [24], who found that in the TA with the therapist in IBIs, “goals and tasks” loaded in the same factor, whereas “bond” loaded in a separate factor. According to our findings, a three-dimensional structure cannot be assumed a priori in the context of IBIs. More specifically, in the case of the TA with an online program during a self-guided IBI, the theoretical distinction between task, goals, and bond with the online program was not psychometrically significant, and a single factor could explain the majority of the explained variance of the TA between the patient and the online program. However, these results should be interpreted with caution because IBIs are continuously evolving, and a more personalized treatment that uses algorithms to provide personalized feedback or set individualized goals or tasks depending on the emotional state or unique needs of each patient throughout the treatment could generate a more differentiated factorial structure of the WAI-TECH-SF.

Another possible explanation for the structure of the WAI-TECH-SF is related to the fact that the TA with the online programs is highly complex, and merely replacing the words is not sufficient to capture the subtle differences in these different kinds of TA. In other words, perhaps the dimensions of the questionnaire should be completely reframed [28]. In this regard, Henson, Peck, and Torous [49] developed the Digital-WAI (D-WAI), a six-item self-report questionnaire based on Bordin’s three dimensions, but aligned with the purpose of smartphone-based interventions (e.g., “bond” is aimed at measuring the capacity of the app to offer support and guide them through challenges). More recently, Miloff et al. [50] adopted this approach of developing novel items and validated the Virtual Therapist Alliance Scale (VTAS), which assesses the three components of the TA with virtual therapists in an automated exposure treatment format for patients with fear of spiders. Two factors emerged in the exploratory factor analysis (“task, goal, and copresence” and “bond and empathy”) that had small and non-significant correlations with therapeutic outcomes at post-treatment, but moderate and significant correlations at follow-up.

Regarding the differences in the WAI-TECH-SF scores according to different characteristics of the sample, overall, no differences were found. That is, the TA with the self-guided IBI was achieved by the patients independently of their sex, their age, the severity of their depression before starting the intervention, their preferences for doing the intervention in the assigned condition, or the expectations and credibility towards the treatment. The average score on the WAI-TECH-SF was around 58 (on a scale ranging from 12 to 84). Nevertheless, patients with a higher level of education scored higher on TA with the online program than patients with a low level of education. This finding was also corroborated by the exploratory multiple regression analysis, in which all the different characteristics of the sample were introduced as potential predictors of the WAI-TECH-SF scores. Results showed that level of education, but also the age, were positive significant predictors of the TA with the self-guided IBI, explaining 4.9% of the variance. Regarding level of education, this higher TA could be related to the fact that more positive therapeutic outcomes in IBIs are also predicted by having a higher level of education [51]. Moreover, these findings may be associated with the lower preferences for IBIs expressed by people with a lower level of education [52], or the related barriers to the use of a less-known technology (e.g., low trust and lack of confidence in the capacity of IBIs to actually help).

The lower preference for technology adoption has also been related to age (e.g., because of their lower proficiency). Moret-Tatay et al. [53] found that older adults showed lower scores in mobile device and computer proficiency than younger adults. Consequently, adapted computer systems for older people have been designed to reduce the barriers that this population encounter. Mitzer et al. [54] found that the use of an adapted computer system for older people at the mid- and long-term was predicted by the earlier use of the system, the higher cognitive abilities (i.e., executive functioning), and computer efficacy. Hence, future studies should assess technology proficiency and cognitive abilities before starting a self-guided IBI in order to avoid the problems associated with the level of education and age, such as the adherence to the therapy. Nevertheless, older patients achieved higher TA in our study. One possible explanation for this finding is that the lower technology proficiency typically found in the population could have been compensated by the greater involvement in the therapy.

Regarding the capacity of the TA with the self-guided IBI to predict therapeutic outcomes, the findings highlight the importance of considering the WAI-TECH-SF scores to predict the change in depressive symptoms and satisfaction with the intervention. The TA with the online program explained 6.7% of the change in depressive symptoms, and 49.7% of the satisfaction with the treatment. Consequently, the relationship between “patient-online program TA” and therapeutic outcomes is also in line with the positive relationship found between the “patient-therapist TA” and the therapeutic outcomes in face-to-face therapy [1,2] and IBIs [3,21]. However, to our knowledge, this is the first study to confirm the relationship between “patient and online program TA” and therapeutic outcomes. By contrast, Kiluk et al. [29] did not find that the total scores on the long form of the WAI-Tech were associated with the change in therapeutic outcomes. Hence, so far, only the present study and Miragall et al. [25] found a significant relationship between the TA with the technology (i.e., the TA between the patient and virtual and augmented reality) and therapeutic outcomes. Therefore, this finding supports the need to work directly on the TA when it is poor because it has important consequences for therapeutic outcomes. Future studies should include algorithms to detect low TA scores after each session, in order to adjust the goals, tasks, and bond between the patient and the online program during an IBI.

This study has some limitations. First, the WAI-TECH-SF was only administered at the end of the treatment, which did not allow us to explore whether the “patient and online program TA” preceded the symptoms and satisfaction throughout the therapeutic sessions. Thus, having these measures during the treatment would allow us to establish the causal effect of TA on the therapeutic outcomes. Future studies should administer the WAI-TECH-SF in earlier therapeutic sessions (e.g., third session) in order to examine the TA through the therapy. Second, the study sample was only composed of depressive patients. Therefore, future studies should replicate this study in a sample of patients with several diagnoses (e.g., anxiety, post-traumatic stress disorder) in order to confirm whether the same psychometric structure is found, and to detect its capacity to predict therapeutic changes in other mental disorders. Third, the adherence or number of sessions performed by the patients was not registered. Thus, future studies should analyse whether the TA affects adherence and, in turn, the therapeutic outcomes. Fourth, the statistical analyses of TA were only conducted with the patients that accepted to fill in the questionnaire after the self-guided IBI was finished. However, the normal distribution (e.g., skewness = −0.56; kurtosis = −0.83) and the wide range of variability of the WAI-TECH-SF scores (i.e., from 12 to 84) allowed us to draw reliable conclusions. The importance shown by the TA with the technology points out the question regarding the impact of TA at early stages of the treatment, and the role that it can play in predicting efficacy and preventing dropouts.

Finally, the importance of having self-guided IBI that promotes an adequate TA between the patient and the online program should be noted, especially when resources are scarce. Several situations, such as the COVID-19 pandemic, could prevent individuals from accessing the traditional face-to-face therapy. Consequently, CBT delivered through telehealth services are undeniably crucial in order to provide timely psychological support, especially in vulnerable populations [55].

## 5. Conclusions

In conclusion, this study reveals that patients with major depression can develop TA with an online program during a self-guided IBI in the context of primary care. Thus, patients can feel that the program is “taking care” of them, in terms of allowing them to achieve therapeutic goals, proposing appropriate tasks to achieve these goals, and making them feel “embraced” and “cared for” by the program. According to our exploratory factor analysis, the WAI-TECH-SF is a reliable questionnaire to measure this construct, but it would be advisable to calculate an overall score for the total scale, rather than using the traditional theoretical three-dimensional “task-goals-bonds” structure of TA. Moreover, it would be beneficial to explore the IBI preferences of the patients with lower education levels before starting the intervention, in order to ensure that their level of education does not interfere with their capacity to develop TA with the online program. Finally, this study highlights the importance of considering the “patient and online program TA” because the WAI-TECH-SF score was a significant predictor variable of both the change in depressive symptoms and satisfaction at the end of the treatment. Further research is needed to more deeply understand the TA achieved in the “patient-technology-therapist” triangulation in blended treatments.

## Figures and Tables

**Table 1:** Overview of blended treatment applied in each country.

					Mandatory Modules				Additional Modules		
Country	Platform	Duration	Online/ Face-to-Face Sessions	Session Sequence	PE	CR	BA	RP	Problem Solving	Physical Exercise	Other
Netherlands	Moodbuster	20 weeks	10/10	Alternate	X	X	X	X	X	X	
France	Moodbuster	16 weeks	8/8	Alternate	X	X	X	X	X	X	
Poland	Moodbuster	6–10 weeks	6/6	Alternate	X	X	X	X	X	X	
United Kingdom	Moodbuster	11 weeks	5/6	Alternate	X	X	X	X	X	X	
Switzerland	Deprexis	18 weeks	9/9	Alternate	X	X	X	X	X	X	X
Sweden	Itherapi	12 weeks	8/4	Alternate	X	X	X	X			
Spain	Smiling is fun	10 weeks	8/3	1-4-1-4-1	X	X	X	X			X
Germany	Moodbuster	10–13 weeks	10/5	Alternate	X	X	X	X	X	X	

Notes. PE = Psychoeducation; CR = Cognitive restructuring; BA = Behavioral activation; RP = Relapse prevention.

**Table 2:** Psychometric properties of the WAI-TECH: Descriptive statistics and factorial loadings with a one-factor structure using Maximum Likelihood.

	Skewness Index	Kurtosis Index	M (SD)	*λ*	*h* ^2^
**Item 1.** As a result of these sessions using the program____ I am clearer as to how I might be able to change.	−0.72	0.19	5.11 (1.40)	0.76	0.58
**Item 2.** What I am doing with the program____ gives me new ways of looking at my problem.	−0.56	−0.40	4.89 (1.48)	0.88	0.77
**Item 3.** I believe that I am a good candidate for the program___.	−0.50	−0.36	4.84 (1.46)	0.86	0.74
**Item 4.** The program___ and I collaborate on setting goals for my therapy.	−0.47	−0.58	4.72 (1.72)	0.87	0.75
**Item 5.** The program___ and I respect each other.	−0.57	−0.28	4.94 (1.58)	0.84	0.70
**Item 6.** The program____ and I are working towards mutually agreed upon goals.	−0.64	−0.05	5.04 (1.48)	0.85	0.72
**Item 7.** I feel that the program____ appreciates me.	−0.35	−0.75	4.44 (1.74)	0.81	0.67
**Item 8.** The program___ and I agree on what is important for me to work on.	−0.62	−0.25	4.84 (1.61)	0.85	0.73
**Item 9.** I feel the program_____ cares about me even when I do things that he/she does not approve of.	−0.61	−0.13	4.91 (1.62)	0.89	0.78
**Item 10.** I feel that the things I do with the program___ will help me to accomplish the changes that I want.	−0.48	−0.34	4.56 (1.54)	0.87	0.76
**Item 11.** The program___ and I have established a good understanding of the kind of changes that would be good for me.	−0.66	−0.07	4.93 (1.55)	0.91	0.83
**Item 12.** I believe the way that the program___ and I are working with my problem is correct.	−0.52	−0.43	4.62 (1.65)	0.89	0.79

Notes. *λ* = Factor loading; *h*^2^ = Communalities.

**Table 3:** Descriptive statistics of WAI-TECH scores in each category.

	Independent-Sample *t*-Tests/ One-Way ANOVAs	N	M	SD
Total sample		193	57.84	16.39
Sex	*t*(191) = 0.49, *p* = 0.627, *Cohen’s d* = 0.07			
Men		69	57.07	15.03
Women		124	58.27	17.14
Age-range	*F*(2,190) = 1.75, *p* = 0.177, η^2^_p_ = 0.02			
18–34		70	55.84	17.09
35–49		66	57.12	17.89
>50		57	61.13	13.14
Level of education	*F*(2,190) = 3.21, *p* = 0.043, η^2^_p_ = 0.03			
Low		24	50.01	15.06
Medium		61	58.72	15.52
High		108	59.08	16.80
Initial severity of depression	*F*(3,189) = 0.91, *p* = 0.436, η^2^_p_ = 0.01			
Mild		21	59.86	16.16
Moderate		64	56.50	16.38
Moderate-Severe		71	56.66	17.03
Severe		37	61.27	15.27
Preference for any of the treatments offered	*F*(2,190) = 1.66, *p* = 0.194, η^2^_p_ = 0.02			
No preference		54	57.78	16.09
Blended		107	56.48	16.09
Treatment as usual		32	62.47	17.53
Expectations towards the treatment	*F*(2,182) = 1.34, *p* = 0.265, η^2^_p_ = 0.02			
Low		34	59.84	15.41
Medium		119	57.92	16.82
High		32	53.47	16.18
Credibility towards the treatment	*F*(2,183) = 0.57, *p* = 0.567, η^2^_p_ = 0.01			
Low		28	56.38	17.19
Medium		126	57.13	15.65
High		32	60.34	19.13

**Table 4:** Simple linear regressions of change in PHQ scores and satisfaction with the treatment.

	R	R^2^	B	SE	β	t
Change in PHQ scores						
Constant			0.186	1.829		
WAI-TECH	0.268	0.072	−0.115	0.030	−0.268	3.797 ***
Satisfaction with the treatment						
Constant			12.929	0.951		13.601 ***
WAI-TECH	0.707	0.497	0.214	0.016	0.707	13.621 ***

Note. Statistical significance: *** *p* < 0.001. PHQ-9 = Patient Health Questionnaire-9; WAI-TECH-SF = Working Alliance Inventory applied to Internet–Short Form. R = Multiple Correlation Coefficient; R^2^ = Coefficient of determination; R^2^ Change = Coefficient of determination Change; B = Unstandardized coefficient; SE = Standard Error; β = Beta coefficient; t = t statistic (estimated coefficient divided by its own SE).
